# Patterns and Drivers of Spontaneous Plant Diversity in Urban Wastelands Across 17 Cities on the Qinghai‐Tibet Plateau

**DOI:** 10.1002/ece3.73521

**Published:** 2026-04-29

**Authors:** Lin He, Zhiwen Gao, Yao Yao, Luyi Lan, Xiaoya Yu, Yanyi Yang, Xinyi Luo, Ruishen Yang, Junwei Wang, Yuandong Hu, Qiong La, Liangjun Da

**Affiliations:** ^1^ Institute of Science and Engineering of Ecology in Arid and Semi‐arid Areas Xi'an University of Architecture and Technology Xian Shaanxi China; ^2^ Yunnan Key Laboratory of Plateau Geographical Processes & Environmental Changes, Faculty of Geography Yunnan Normal University Kunming Yunnan China; ^3^ School of Tourism and Resource Environment, Qiannan Normal University for Nationalities Duyun Guizhou China; ^4^ Shanghai Key Laboratory of Urban Design and Urban Science NYU Shanghai Shanghai China; ^5^ School of Ecological and Environmental Sciences, East China Normal University Shanghai China; ^6^ College of Agriculture and Life Sciences Kunming University Kunming Yunnan China; ^7^ Key Laboratory of Biodiversity and Environment on the Qinghai‐Tibetan Plateau Ministry of Education, School of Ecology and Environment, Xizang University Lhasa China; ^8^ Landscape Architecture Northeast Forestry University Harbin Heilongjiang China

**Keywords:** alpha diversity, beta diversity, habitat quality, spontaneous plant, urbanization

## Abstract

The Qinghai‐Tibet Plateau (QTP) is ecologically significant due to its unique biodiversity and vulnerability to climate change and rapid urbanization. Among its emerging urban habitats, wastelands with relatively low anthropogenic disturbances offer key refuges and stepping stones for spontaneous plants. However, the patterns and drivers of spontaneous plant diversity in these habitats remain poorly understood. To address this, we surveyed spontaneous plant communities across 17 cities on the QTP. We found that native species dominated urban wasteland flora (85.8%), whereas invasive species constituted over half (65.8%) of the non‐native species. The results showed that land‐use legacy effects and environmental filtering shape community assembly. GLMM analyses further reveal that the climatic background constitutes a key factor shaping community diversity variations across the QTP, exerting the most significant influence on species richness, particularly through precipitation and wind speed. Urbanization and habitat quality factors jointly shape the diversity structural characteristics of communities. However, non‐native and invasive species exhibit heightened sensitivity to local habitat quality. Furthermore, differences between communities across all groups were due to species turnover, though the driving factors differed between groups. Native species exhibited stronger overall ecological adaptability, whereas differences between communities of non‐native and invasive species were primarily driven by human disturbance and habitat conditions. These findings underscore the ecological value of urban wastelands on the QTP for biodiversity conservation and invasion management under rapid urbanization and climate change.

## Graphical abstract

The first regional survey of spontaneous plant communities across 17 cities on the Qinghai‐Tibet Plateau shows that natural climatic conditions, especially precipitation and wind speed, are the primary drivers of variation in community diversity, while urbanization and habitat quality jointly structure plant community composition. Species turnover dominated *β*‐diversity, indicating that environmental heterogeneity and spatial succession strongly influence community differentiation in urban wastelands. These findings highlight the ecological importance of QTP urban wastelands for conserving native biodiversity and managing plant invasions under accelerating urbanization and climate change.

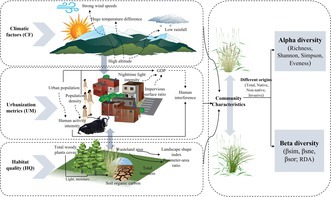

## 1 Introduction

The Qinghai‐Tibet Plateau (here after QTP), often referred to as the third pole of the world, is a biodiversity hotspot and an ecologically sensitive region shaped by harsh climate, high altitude, and a unique biogeographical history. The region supports a distinctive array of plant communities and numerous rare wildlife (Kandel et al. 2019; Pu et al. 2019; Zhang et al. 2002). Recently, the impacts of global climate warming have posed increasing challenges to biodiversity conservation efforts on the Plateau (An et al. 2017; Lamsal et al. 2017; Li, Kraft, et al. 2015). In addition, rapid urbanization over the past few decades has led to profound changes in land use and landscape structures across the region (Li et al. 2018). These transformations have significantly altered key environmental factors, such as temperature regimes, precipitation patterns, light availability, and soil properties, leading to highly heterogeneous urban habitats (Chen et al. 2014; Gao et al. 2023; Zhu et al. 2024). Among the various urban habitats shaped by these changes, urban wastelands are defined as abandoned, unused land within cities and represent the least disturbed type of urban green space (McPhearson et al. 2013). In urban settings, they typically occur on sites such as demolished residential or infrastructural land, post‐industrial areas, abandoned warehouses, and disused railways. In this study, urban wastelands are defined as long‐abandoned open spaces within cities dominated by spontaneous plants (Bonthoux et al. 2014).

Urban wastelands, unlike managed parks, residential areas, or landscaped gardens, experience relatively low levels of direct human intervention. It allows for the persistence of diverse and, in some cases, even provides important refuges for endemic, rare, and endangered species (Ives et al. 2016; Lepczyk et al. 2023). In addition, urban wastelands serve as critical habitats for urban wildlife, contribute to ecological restoration, and function as seed banks to support plant regeneration in other urban green spaces (Javier et al. 2020; Xia et al. 2024). They may also serve as corridors and stepping stones, facilitating species dispersal and gene flow that are essential for maintaining urban biodiversity and ecological stability (Cui et al. 2019; Kattwinkel et al. 2011). Therefore, urban wastelands provide a relatively natural environment within the urban matrix, making them ideal sites for studying the distribution, colonization, and successional dynamics of plants in urban environments (Bonthoux and Chollet 2024; Meffert et al. 2012). Insights gained from these relatively undisturbed habitats can offer valuable references for the management and ecological utilization of vegetation in other types of urban green spaces.

Urban wastelands are primarily colonized by spontaneous plants, which play a central role in shaping their ecological character. Spontaneous plants, also referred to as self‐established species, are a vital component of urban biodiversity (Cervelli et al. 2013; Gao et al. 2025; Jin et al. 2024). Unlike remnant natural vegetation or intentionally cultivated species, spontaneous plants colonize and thrive in almost all urban habitats (Chen et al. 2025; Li et al. 2024; Xu et al. 2025). Their remarkable adaptability and resilience enable them to persist amid frequent disturbances and the rapidly changing environmental conditions characteristic of urban areas, making them ideal for monitoring ecological responses to urban development and disturbance (Pianta et al. 2025; Yuan et al. 2023; Zong et al. 2025). Their presence, abundance, and community composition are highly sensitive to environmental changes. By examining the patterns and drivers of spontaneous plant diversity within and among cities, researchers can gain valuable insights into the processes shaping urban ecosystems and the interactions between plant communities and the urban environment (Zhao et al. 2025).

Although spontaneous plants can establish in almost all types of urban habitats, urban wastelands, where they often dominate, offer a unique opportunity to study their ecological roles under relatively undisturbed conditions (Petit‐Berghem et al. 2021). Studying the diversity of spontaneous plant communities in urban wastelands not only enhances our theoretical understanding of urban ecosystems but also has practical applications. For instance, understanding the natural succession patterns in these relatively undisturbed habitats (Yang et al. 2022), revealing the adaptive strategies and resilience of spontaneous plants in harsh urban environments (Fischer et al. 2013; Kowarik 2023), and recognizing the ecological value of urban wastelands. Ultimately, such studies can promote the adoption of nature‐based solutions that leverage spontaneous plants to address urban environmental challenges and improve the quality of life for city residents (Köppler et al. 2014; Zoderer and Hainz‐Renetzeder 2025).

Nevertheless, urban wastelands are still influenced by the broader impacts of anthropogenic and climate factors. Disturbances such as surrounding land use changes and ongoing urban development can influence species dispersal and community assembly within these wastelands. For instance, the urbanization intensity in adjacent areas can change the immigration of plant propagules (Muratet et al. 2007), thereby shaping the composition and diversity of spontaneous plant communities (Hou, Shi, et al. 2023). Additionally, the length of abandonment time potentially represents the successional stage and establishment of plant communities, with older wastelands supporting higher biodiversity due to longer colonization periods and more developed soil conditions (Li, Cadotte, et al. 2015). Moreover, habitat quality within each wasteland, including factors such as vegetation cover, soil properties, and the degree of impervious surface, further modulates community structure and ecological processes (Godefroid et al. 2007; Javier et al. 2020). At broader spatial scales, regional climatic factors also play a significant role in determining plant diversity in urban wastelands (Petit‐Berghem et al. 2021). Variations in temperature, precipitation, solar radiation, and other bioclimatic conditions can create heterogeneous environmental filters that influence which species can persist and thrive in these habitats.

In recent years, with the rise of urban ecology and low‐maintenance landscaping concepts, spontaneous plants have attracted increasing academic attention. Existing studies mainly focus on diversity patterns (Chen et al. 2025), landscape applications and ecological functions (Hong et al. 2025), influencing mechanisms (Chen and Wang 2025), and public perception and management strategies (Su et al. 2025; de Val 2023). However, several gaps remain. Research on spontaneous plants in urban wastelands most studies are conducted at local scales, focusing on single cities or regions, with a lack of large‐scale, cross‐regional analyses spanning different climate zones and elevation gradients (Guo et al. 2025); Moreover, existing research is largely concentrated in low‐altitude, economically developed cities, while high‐altitude and ecologically sensitive regions such as the QTP remain understudied (Gao et al. 2023; Zong et al. 2025). In addition, few studies have adopted an integrated framework to examine the combined effects of climate, habitat quality, and urbanization on spontaneous plants of different origin types.

In this context, our study investigates spontaneous plants in urban wastelands across 17 cities on the QTP, combining field surveys with environmental data to: (1) clarify the spatial pattern of spontaneous plant diversity in urban wastelands at the regional scale; (2) identify the key drivers, including climatic, urbanization metrics, and habitat quality drivers, that influence plant alpha and beta diversity; (3) quantify the relative contributions of these drivers to the observed diversity patterns. We assess multiple facets of alpha diversity, including species richness (number of species), community complexity and stability (Shannon‐Wiener index), species evenness (Pielou index), and dominant species status (Simpson index). For beta diversity, we examine variation in community composition by decomposing overall *β*‐diversity into species turnover (*βsim*) and nestedness (*βsne)* components using the Sørensen index (*βsor*). Through this integrated approach, the study aims to advance a more comprehensive understanding of urban biodiversity in high‐altitude environments.

## 2 Materials and Methods

### 2.1 Study Area

**FIGURE 1:**
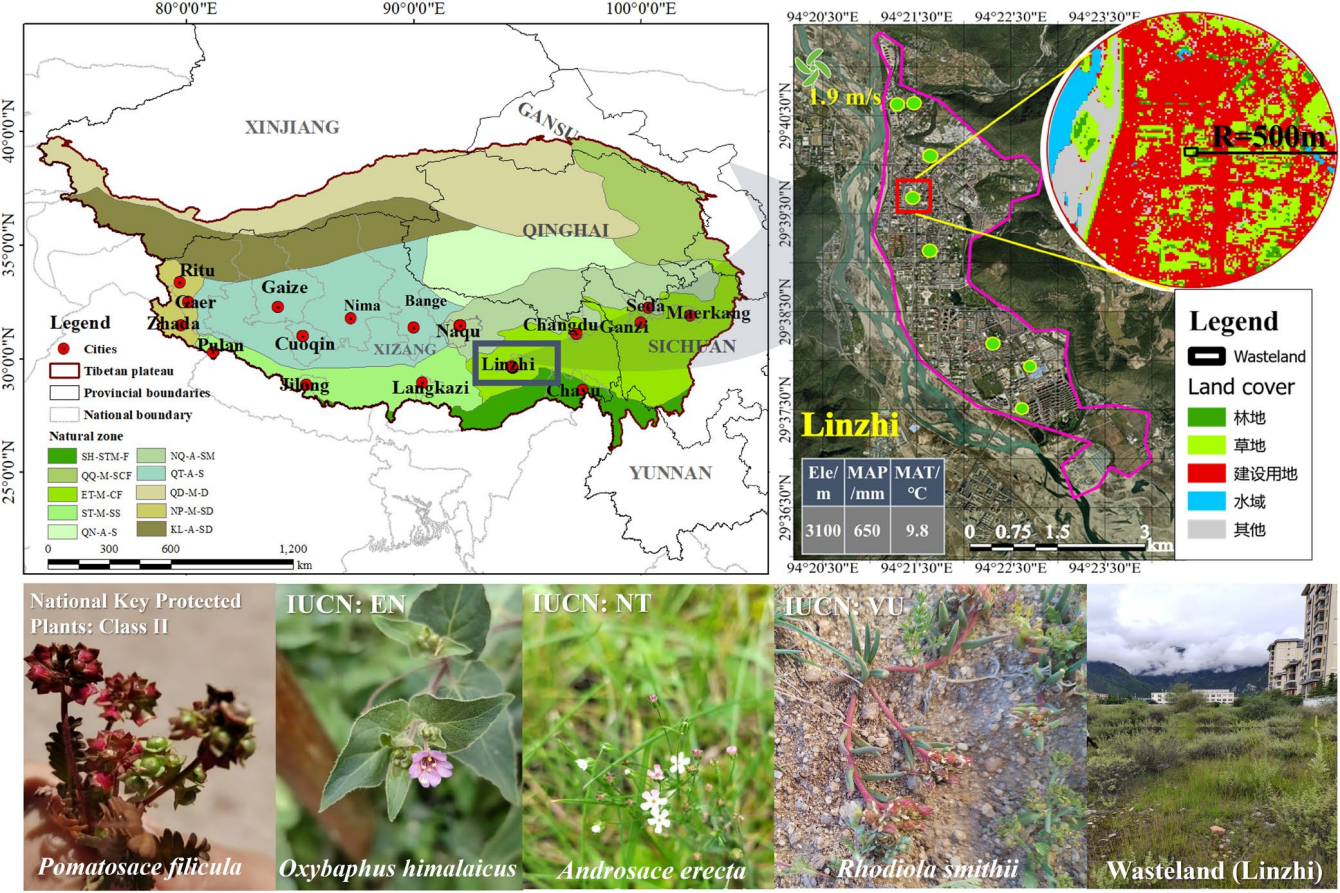
Overview of the 17 surveyed cities on the QTP. A, alpine; CF, coniferous forests; D, desert; F, forests; M, mountainous; NGZ, natural geographic zones; S, steppe; SD, semi‐desert and desert; SM, shrub‐steppes; SS, shrub‐steppe; ST, subtropical and tropical (abbreviation based on first letter). In each city, the pink polygons represent the boundaries of the built‐up area, and the green dots represent the locations of the surveyed wastelands, using Linzhi as an example. Top right: Land use classification map within the built‐up area of Linzhi, centered on one of the wastelands with a radius of 500 m (Kappa: 0.85; P_Accuracy: 0.88). Bottom left: A selection of rare, endangered, and protected plant species identified in the survey; bottom right: Photographs of the wastelands. All photographs of plants and wasteland in the images were taken by the authors.

The QTP (25°59′30″ N~40°1′0″ N, 67°40′37″ E~104°40′57″ E), with a total area of more than 2.5 million square kilometers and an average elevation of approximately 4320 m, is the highest plateau in the world (Figure 1). Rich in biological resources, it is recognized as one of the biodiversity hotspots in China, making it a region of high biodiversity conservation and research (Zhang et al. 2002, 2021). However, plants on the QTP face multiple environmental challenges, including high altitude, low precipitation (mean 482.80 mm/year), intense solar radiation (mean 2323 h/year), and strong wind speed (mean 2.35 m/s), which pose considerable threats to biodiversity (Jiang et al. 2015; Ye et al. 2022). Since the 1980s, cities on the QTP have been experiencing a rapid urbanization process, including infrastructure development and population growth, making the impacts of urbanization on biodiversity in the region increasingly prominent and worthy of attention. According to the results of the national census, the resident population of the region has risen from 8.43 million people in 1982 to 13.13 million people in 2020 (Fang 2022). Meanwhile, the region's economy is growing rapidly, with gross domestic product (GDP) increasing about 20‐fold over the past three decades (Xu 2022; Xu et al. 2017).

In this study, to comprehensively reflect the community characteristics of urban wastelands on the QTP, we selected 17 cities across the QTP, covering a wide range of natural environmental gradients, including altitude (2310–4722 m), mean annual precipitation (0–1063 mm), mean annual temperature (0.31°C–11.67°C), mean annual wind speed (1.59–4.39 m/s) and annual sunshine hours (1686–3612 h). Urbanization levels also differ considerably, as reflected by urban population size (2200–50,000 people) and urban area (0.99–18.04 km^2^). Collectively, these cities provide a representative sample of the region's environmental heterogeneity and urban development spectrum.

### 2.2 Data Collection

#### 2.2.1 Field Surveys

Environmental and socioeconomic variables, including climate, elevation, and economic development level, were extracted for each city. Subsequently, stratified sampling was conducted using the *survey* package in *R* to select cities that comprehensively represent gradients in natural and socioeconomic conditions across the region. Field surveys were conducted during the plants' growing season (July–August, 2024) across urban wastelands of the QTP. Within the urban built‐up area, before conducting the survey, we conducted a preliminary identification and delineation of potential wastelands using high‐resolution remote sensing imagery (Google Earth), identifying between 3 and 12 potential wastelands. Then, we verified each wasteland through on‐site inspections and, taking accessibility into account, finalized the selection of the wastelands. We conducted community surveys on the selected accessible wastelands. Eventually, 41 wastelands in 17 cities were selected. During field surveys, each wasteland was first comprehensively assessed, and plots were established in representative plant communities with vegetation cover ≥ 30% to ensure adequate representation of the diversity of community types and their compositional characteristics within each site. In total, 590 1 × 1 m herbaceous plots were surveyed (Table S1). Following the *Guidelines for the Classification of Land and Marine Use in National Territorial Space Surveys, Planning, and Land‐Use Control (2023)*, the land‐use types of the wasteland before abandonment were classified into the following categories: cultivated land (CL), residential land (RL), storage land (SL), green and open space (GOS), industrial and mining land (IML), special‐use land (SUL, military land, etc.), land‐based water bodies (WB), and other land (OL, including fallow land, bare land, sandy land, and reserve arable land) (Table S2).

Within these plots, the maximum height of each species, species cover, maximum community height, and total community cover were recorded. We classified plant species based on their life forms and origin types. According to Wu (1980), plants were categorized into woody plants and herbaceous plants, the latter further divided into perennials and annuals. Plant species were classified as native, non‐native, and invasive origin type groups based on the origin types provided by Ma (2013) and Lin et al. (2022). These references provide comprehensive assessments of plant species origins in China. Specifically, species historically distributed within the QTP or adjacent native floristic regions were classified as native. Species that have departed from their native range through natural or anthropogenic factors (whether intentionally or unintentionally introduced) and entered other territories (typically referring to countries or regions), and are capable of surviving and persisting on the QTP were identified as non‐native (Lin et al. 2022). Invasive plants refer to non‐native taxa among the aforementioned species that, during their establishment and growth, exhibit rapid spread and high ecological competitiveness, or exert detrimental or beneficial effects upon native flora. Such impacts may be temporarily advantageous or neutral, significant or hidden, and ultimately alter or threaten the biodiversity of the region (Ma 2013).

#### 2.2.2 Environmental Variables

**FIGURE 2:**
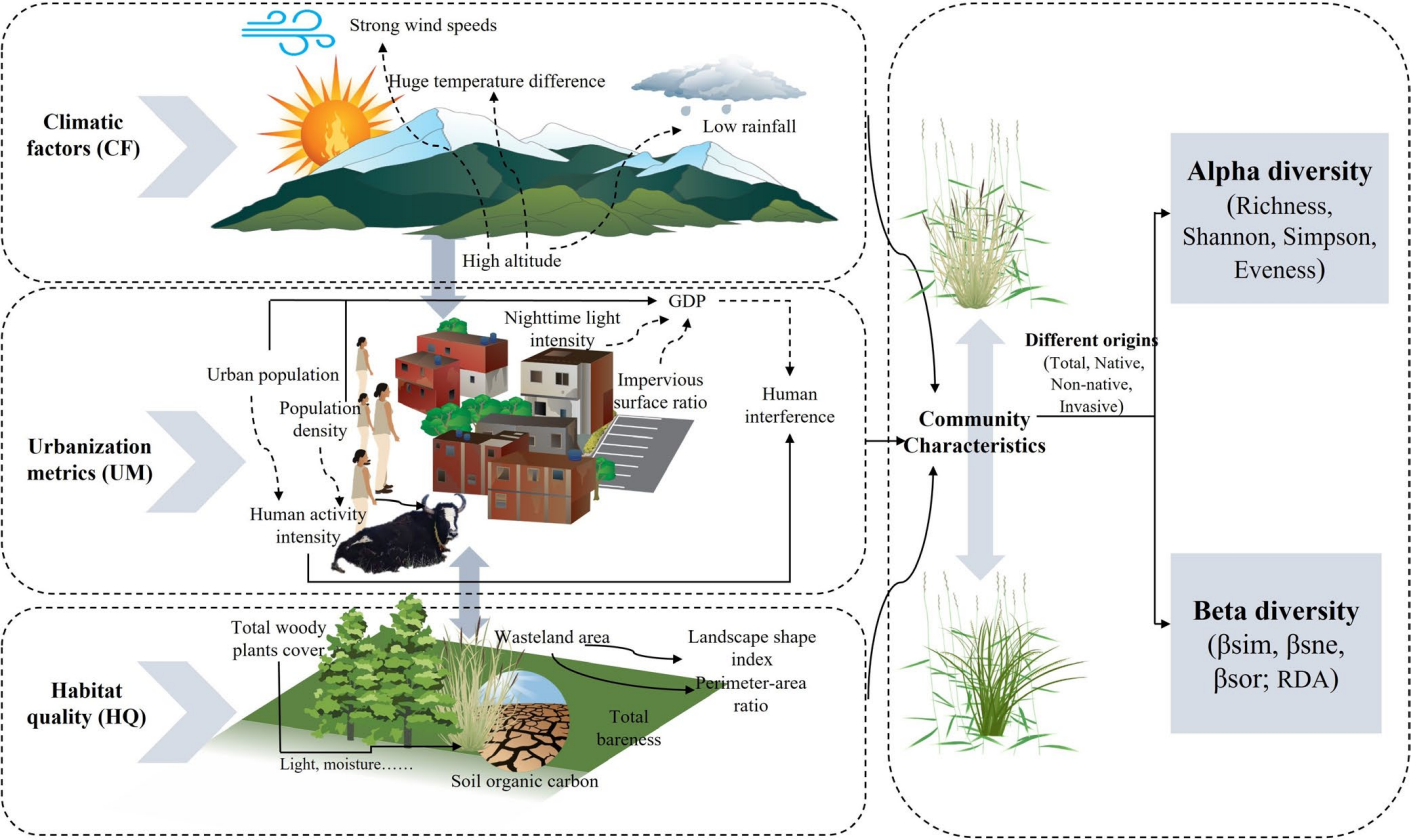
Conceptual diagram of the impact of different drivers on urban biodiversity (Icons used in this figure were obtained from the Integration and Application Network [IAN] image library [https://ian.umces.edu/media‐library/], University of Maryland Center for Environmental Science, and are used under their free‐use policy for academic purposes. Arrows and figure layout were created by the authors using Microsoft PowerPoint).

This study constructs a comprehensive framework incorporating three dimensions—climatic stress, urban disturbance, and habitat filtering—to select environmental variables, based on an understanding of biodiversity drivers within urban ecosystems. We hypothesize that plant community diversity is simultaneously governed by regional climate patterns, directly influenced by anthropogenic disturbances and resource gradients associated with urbanization, and filtered by local habitat characteristics and spatial structure. The selected variables are designed to quantify key ecological processes across these three core dimensions (Figure 2).

Therefore, environmental variables, including climatic factors, urbanization metrics, and habitat quality, were collected to assess the drivers of spontaneous plant species diversity in urban wastelands (more details please see Table 1). We consider two primary factors from classic climate characteristics: temperature and moisture. Additionally, we account for the unique conditions of high altitudes. Therefore, our climatic indices include: Mean annual precipitation (MAP), mean annual temperature (MAT), total annual solar radiation (TAR), and mean annual wind speed (MAW). Urbanization intensity was represented by seven metrics, including gross domestic product (GDP), GDP per capital (RGDP), and population density (PD), human activity intensity (HAI), urban population (UP), impervious surface ratio within a 500 m buffer surrounding each wasteland (Sealed.wl, it reflects the land‐use patterns surrounding the wasteland and the impact of edge effects, thereby providing an indication of the availability of gene pools for species dispersal and escape from the site), and night light intensity (NLI). As for habitat quality, we used seven indicators derived from field surveys and spatial analysis, including factors that directly influence the plant diversity of wasteland: Total woody plants cover in each wasteland (TWC) is defined as the sum of the cover of all woody plant species within a given wasteland (non‐mutually exclusive canopy overlap), calculated as the proportion of the area occupied by woody plants (primarily including shrubs, trees, and woody vines) relative to the total wasteland area, which reflects the overall dominance of woody vegetation and, to a certain extent, the baseline natural conditions of the area. It also reflects vegetation structure and habitat complexity, associated with ecological stability and biodiversity; Total bareness in each wasteland (TB), which serves as a proxy for disturbance intensity and habitat degradation. By analyzing land‐use changes at each abandoned site using historical versions of Google Maps (1984–2025), it is possible to estimate the time of abandonment (AT). The study of wasteland covered a continuous gradient ranging from the early stages of abandonment (< 2 years) to the late stages of abandonment (exceeding 20 years). This indicator can serve as an indicator of the successional stage and ecological recovery potential; Wasteland area (W.area) reflects the size of the habitat island; Soil organic carbon content in the 0–30 cm layer (Ct) reflects the soil quality of wasteland. It also includes factors that indirectly affect biodiversity: Perimeter‐area ratio of wasteland (PA, PA = Wasteland perimeter/Wasteland area, the area and perimeter of the wasteland were calculated using ArcGIS 10.7) and landscape shape index (LSI), which indicates shape regularity and geometric complexity of wastelands.

**TABLE 1:** Description of explanatory variables used in the models to explain plant diversity of spontaneous plant communities.

Category	Variable/Unit	Description	Resolution	Ecological significance	Source
Climatic factors (CF)	MAP (mm)	Mean annual precipitation	1 km	Fundamental physiological filter: Hydrothermal conditions constitute the fundamental determinant of plant survival and represent a major large‐scale driver of species distribution ranges	*Resource and Environmental Science and Data Platform*; https://www.resdc.cn/ (Xu 2022)
	MAT (°C)	Mean annual temperature	1 km		
	MAW (m/s)	Mean annual wind speed	1 km	Wind speed and solar radiation intensity are significant environmental stress factors at high altitudes: wind influences transpiration, mechanical damage, and pollination; radiation directly impacts photosynthesis and energy input	
	TAR (MJ/m^2^)	Total annual solar radiation	1 km		
Urbanization metrics (UM)	GDP (10^4^ CNY/km^2^)	Gross domestic product	1 km	Direct regional economic intensity: This characterizes the overall economic activity within a region, reflecting the level of resource input, while indirectly influencing habitats	https://www.resdc.cn/ (Xu 2020a)
	RGDP (%)	GDP per capital, RGDP = GDP/Population	1 km		
	UP (10,000 persons)	Urban population	/	Directly and indirect reflects the urban clustering effect: urban population characterizes the scale of regional urbanization; nighttime lighting has been extensively demonstrated to correlate strongly with energy consumption, and building density	2022 China Statistical Yearbook of Urban Construction and 2022 China Statistical Yearbook of County City Construction. https://www.geodoi.ac.cn/ (Zhong et al. 2022)
	NLI (DN value)	Nighttime light intensity	0.5 km		
	PD (persons/km^2^)	Population density	1 km	Direct human disturbance intensity: High population density is often accompanied by more frequent human trampling and other activities, exerting direct pressure on biodiversity; HAI typically integrates multiple pressures such as construction and transportation, effectively capturing the degree of habitat disturbance	https://www.resdc.cn/ (Xu 2020b)
	HAI (/)	Human activity intensity	1 km		*National Qinghai‐Tibet Plateau Science Data Center*, https://data.tpdc.ac.cn/ (Liu 2023)
	Sealed.wl (%)	Impervious surface ratio, Sealed.wl = Sealed area/Total area of a 500 m buffer surrounding each wasteland	/	Local habitat fragmentation and isolation: This metric directly quantifies the degree to which wasteland is surrounded by impervious surfaces, directly impacting species dispersal, gene flow, and disturbance isolation	ArcGIS10.7
Habitat quality (HQ)	TWC (%)	Total woody plants cover, TWC = Area covered by woody plants/Total area of the wasteland	/	Habitat complexity and resource competition: The canopy cover of woody plants directly influences light availability and the formation of shaded environments, serving as a key indicator of habitat structure and successional stage	Field survey
	TB (%)	Total bareness, TB = Bare area/Total wastelands area	/	Indirectly reflects disturbance conditions and soil habitat conditions: The proportion of bare ground provides settlement opportunities for pioneer species and annual plants, serving as an indicator of natural or human‐induced disturbance while also reflecting soil quality to some extent	
	Ct (kg C/m^2^)	Soil organic carbon content in the 0–30 cm layer	1 km	Soil fertility and ecosystem function: Soil organic carbon is central to soil health and nutrient cycling, directly influencing plant growth	*National Qinghai‐Tibet Plateau Science Data Center*, https://data.tpdc.ac.cn/(Liu 2023; Wang 2025)
	W.area (ha)	Wasteland area	/	Species‐area relationship: Habitat area is the most fundamental ecological law determining species abundance	Google Earth
	AT (years)	Abandonment time	/	Ecological succession time: Abandonment time is a key temporal factor driving the natural succession of plant communities, directly determining the developmental stage and species composition of the community	Historical Google Earth map (1984–2025)
	PA (m/m^2^)	Perimeter–area ratio, PA = Wasteland perimeter/Wasteland area	/	Edge effects and disturbances: A higher perimeter‐to‐area ratio, indicating more complex shapes, typically signifies richer microhabitats and edge habitats, but may also be subject to greater external disturbances	ArcGIS10.7
	LSI (/)	Landscape shape index, LSI = $\mathrm{WP}/2\sqrt{\pi *\mathrm{WA}}$ (WP = Wasteland perimeter, WA = Wasteland area)	/		

### 2.3 Data Analysis

Species richness directly quantifies the pool of native versus invasive taxa, addressing our core question of how climate, urbanization, and habitat structure shape wasteland communities. The Shannon–Wiener index weights rare species more heavily, capturing early‐stage invasions and the persistence of uncommon native specialists under environmental stress. The Simpson index emphasizes dominant species and reveals whether communities are controlled by a few aggressive invaders or maintain balanced competitive hierarchies. Pielou's evenness isolates equitability independent of richness, distinguishing functionally diverse assemblages from those dominated by few species—a key contrast when evaluating invasion impact and native resilience. Together, these four complementary metrics capture the full spectrum of community structure—from species accumulation to compositional balance and dominance—ensuring we do not rely on a single, potentially misleading summary. The following four diversity indices were selected as community diversity indices, and the calculation formula of each index is:

1. Species richness (Richness) (Whittaker 1972)

(1) Richness=S

2 Shannon‐Wiener diversity index (Shannon) (Shannon 1948)

(2) $$\text{Shannon}=-\sum P_{i}\ln P_{i}$$

3 Simpson diversity index (Simpson) (Simpson 1949)

(3) $$\text{Simpson}=1-\sum {P_{i}}^{2}$$

4 Pielou‐evenness index (Pielou) (Pielou 1966)

(4) $$\text{Pielou}=-\sum P_{i}\ln P_{i}/\ln S$$

In the above equation, *S* is the number of plant species in the plot, $P_{i}$ is defined as the relative importance of the *i*th species ($P_{i}=\frac{H_{i}*C_{i}}{\sum H_{i}*C_{i}}$, *H*_*i*_ is the height of the *i*th species, *C*_*i*_ is the cover of the *i*th species).

This study used the Gleason index (Gleason index, Gleason index=Richness/lnA, *A* = Total area of the plots) to explore the species type changes among different cities. To compare differences in four spontaneous plant diversity among land‐use types, Kruskal–Wallis test was employed. When significant differences were found, post hoc pairwise comparisons were conducted using the *Dunn's test* with *Bonferroni* correction.

Before model fitting, predictors were standardized by using the *scale* function in the *base* package to mitigate scale disparities. Then, a generalized linear mixed model (GLMM) was performed by using independent variables (climatic factors, urbanization metrics, and habitat quality) and nested random intercepts (CityID/WastelandID) with each dependent variable (four plant diversity indexes of total, native, non‐native, and invasive species groups). Since richness is a count variable, the Poisson distribution was used by the g*lmer* function of the *lme4* package; the Gaussian distribution was used for other continuous data by using the *lmer* function of the *lme4* package, and due to zero inflation in the non‐native and invasive groups, the zero‐inflation model was selected by using the *glmmTMB* package with the *glmmTMB* function (Bates et al. 2015; Brooks et al. 2017). After constructing the model, the variance inflation factor (VIF) of the model was further examined by using the *vif* function in the *car* package, and variables with VIF > 5 were removed from the model one by one until all variables had VIF values < 5 (James et al. 2014; Scott 1997; Vittinghoff et al. 2005).

We employed full subset model selection using the *dredge* function from the *MuMIn* R package, and identified the best model based on the corrected Akaike Information Criterion (AICc). When multiple models fell within ΔAICc < 2 of the best model, model averaging was performed using the *model.avg* function to account for model uncertainty (Anderson and Burnham 2004). To quantify the individual explanatory power of each fixed effect, we decomposed the marginal *R* ^2^ ($R_{m}^{2}$) using the *r.squaredGLMM* function in the *MuMIn* R package, allowing us to estimate the relative contribution of each predictor to the overall model performance (Gao et al. 2023; García‐Palacios et al. 2018). All the above calculations were performed in R 4.4.2 software (R Core Team 2023).

To investigate the driving mechanisms behind community composition differences, we first decomposed overall β‐diversity into species turnover (*βsim*) and nesting components (*βsne*) using the Sørensen *β*‐diversity index (βsor) (Baselga 2010). *β*‐diversity calculations were performed using the R package *betapart*, with the *beta.pair* function generating three index matrices—*β* *sor*, *β* *sim*, and *βsne*—for species of different origins. Subsequently, to assess the explanatory power of environmental variables on spatial variation in β‐diversity, we employed Redundancy Analysis (RDA) based on distance matrices (Legendre and Gallagher 2001). RDA analysis was implemented using the *vegan* package. The *rda* function calculated constrained axes, and model significance was assessed via permutation tests (*n* = 999) using *anova.cca*. This further quantified the independent contributions of each environmental factor to community variation.

## 3 Results

A total of 268 plant species (43 families, 160 genera) was recorded across 590 plots in 17 QTP cities. The flora was strongly dominated by native species (*Suaeda corniculate* and *Krascheninniko* via *ceratoides*) (85.8%), while 14.2% were non‐native, among which 65.8% were invasive ( *Erigeron canadensis* and *Dysphania ambrosioides* ). In addition to the dominance of native species, our survey also recorded some rare and endangered taxa (Ministry of Environmental Protection of the People's Republic of China, Chinese Academy of Sciences Press 2013), including *Rhodiola smithii* and *Hippophae salicifolia*. We further identified nationally protected species (National Forestry and Grassland Administration 2021), such as the second‐class protected plant *Pomatosace filicula*, as well as several Tibetan endemic species (Wu 1980), including *Spiraea lobulata*. In total, more than 20 such species were documented across the surveyed urban wastelands (Figure 1).

**FIGURE 3:**
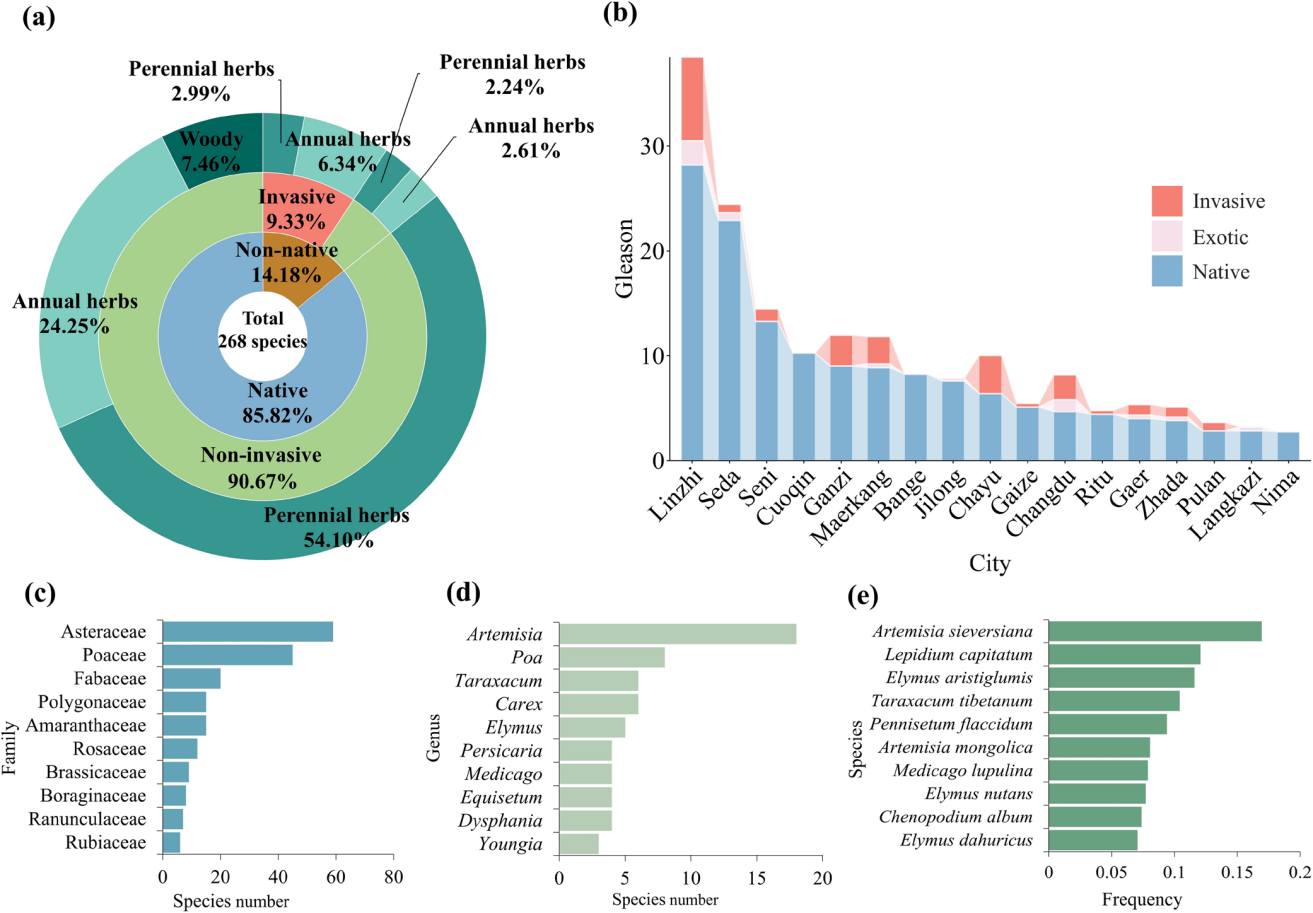
Composition of spontaneous plants in 17 cities of QTP. (a) life form and origin type composition (native, non‐native, invasive); (b) Gleason index in different cities; 10 most prevalent families (c), genera (d), and species (e).

Herbaceous plants made up the vast majority (92.5%). In most of the surveyed areas, only herbaceous plants were found (such as Bange and Nima), with 64.1% being perennial herbs and 35.9% annuals (Figure 3a). Woody plants were mainly found in areas with favorable natural conditions, such as Linzhi and Changdu. Gleason index values ranked from 28.18 (Linzhi) to 2.73 (Nima) revealed a consistent dominance of native species across all surveyed cities. Although no non‐native species were recorded in three cities (Cuoqin, Bange, and Nima), it is noteworthy that in all other surveyed locations, invasive species accounted for over 79.1% of the non‐native flora, highlighting the widespread presence and dominance of invasive taxa wherever introductions had occurred (Figure 3b).

The top three frequent families included Asteraceae (59 species, 22.0%), Poaceae (45, 16.8%), and Fabaceae (20, 7.5%), with the top 10 families accounting for more than 70% of the total species (Figure 3c). At the genus level, *Artemisia* (18, 6.7%), *Poa* (8, 3.0%), and *Taraxacum* (6, 2.2%) were most frequent (Figure 3d). The 10 most common species were all native herbaceous plants, led by *Artemisia sieversiana* (101 plots, 16.9%), followed by *Lepidium capitatum* (72, 12.1%), and *Elymus aristiglumis* (69, 11.6%) (Figure 3e).

**FIGURE 4:**
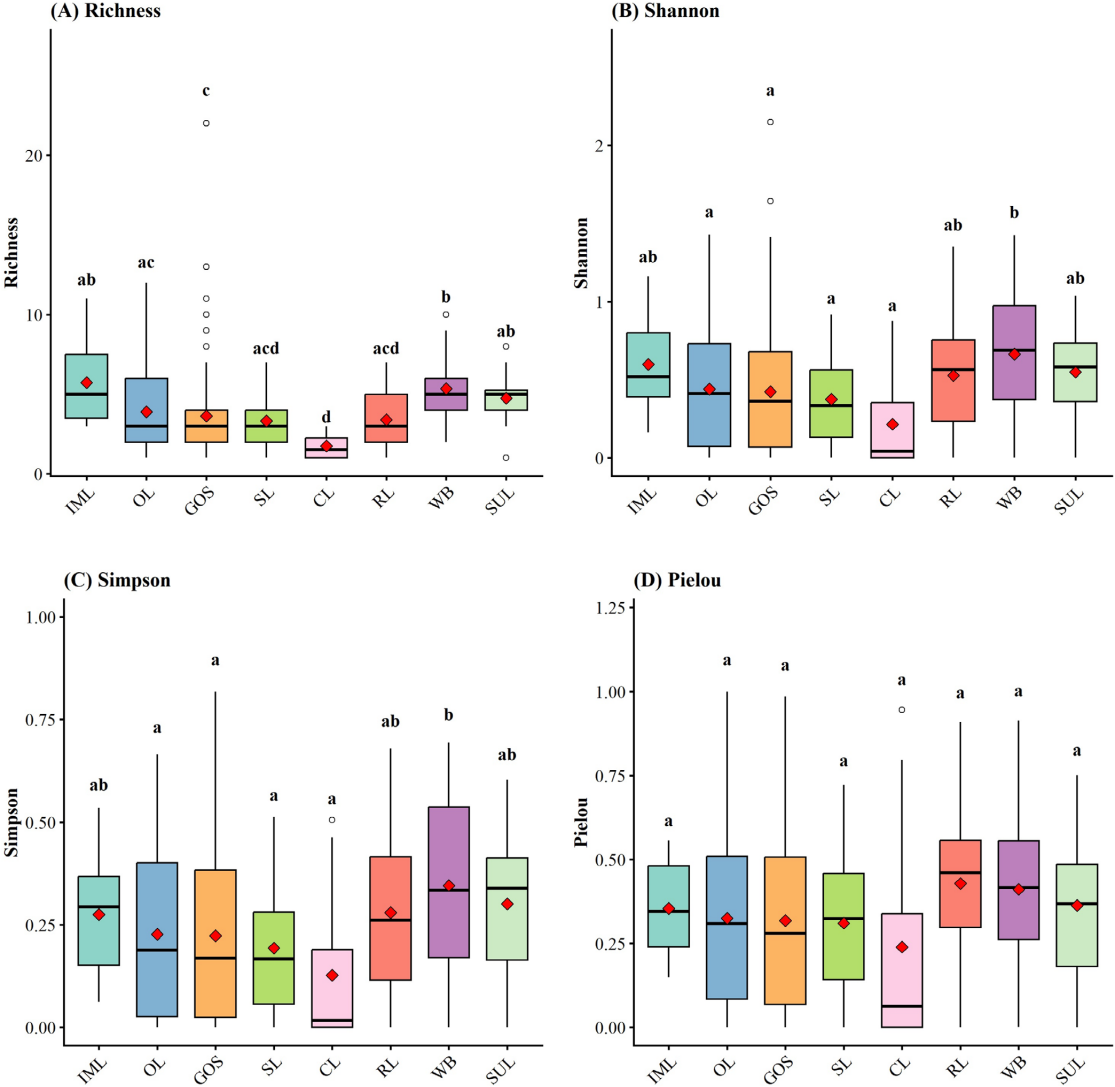
Impact of different types of wastelands on spontaneous plant diversity. (A) Species richness; (B) Shannon index; (C) Simpson index; (D) Pielou evenness. CL, cultivated land; GOS, green and open space; IML, industrial and mining land; OL, other land; RL, residential land; SL, storage land; SUL, special‐use land; WB, land‐based water bodies. Different lowercase letters indicate significant differences among groups at *p* < 0.05.

The different origins of wasteland have a significant impact on species richness, the Shannon and the Simpson, but no apparent effect on the Pielou. This suggests that historical legacy effects primarily screen the number of species colonizing the site by altering environmental thresholds, without altering the relative abundance distribution patterns among species within the community. The convergent evenness across land types reflects similar stages of succession or shared environmental filtering mechanisms within the wasteland habitats. Specifically, although WB exhibited the highest average species richness (5.4) and diversity indices (Shannon = 0.66, Simpson = 0.35), there were significant differences (*p* < 0.05) in species richness and diversity indices (Shannon and Simpson indices) compared with CL, SL, and OL (Figure 4).

**FIGURE 5:**
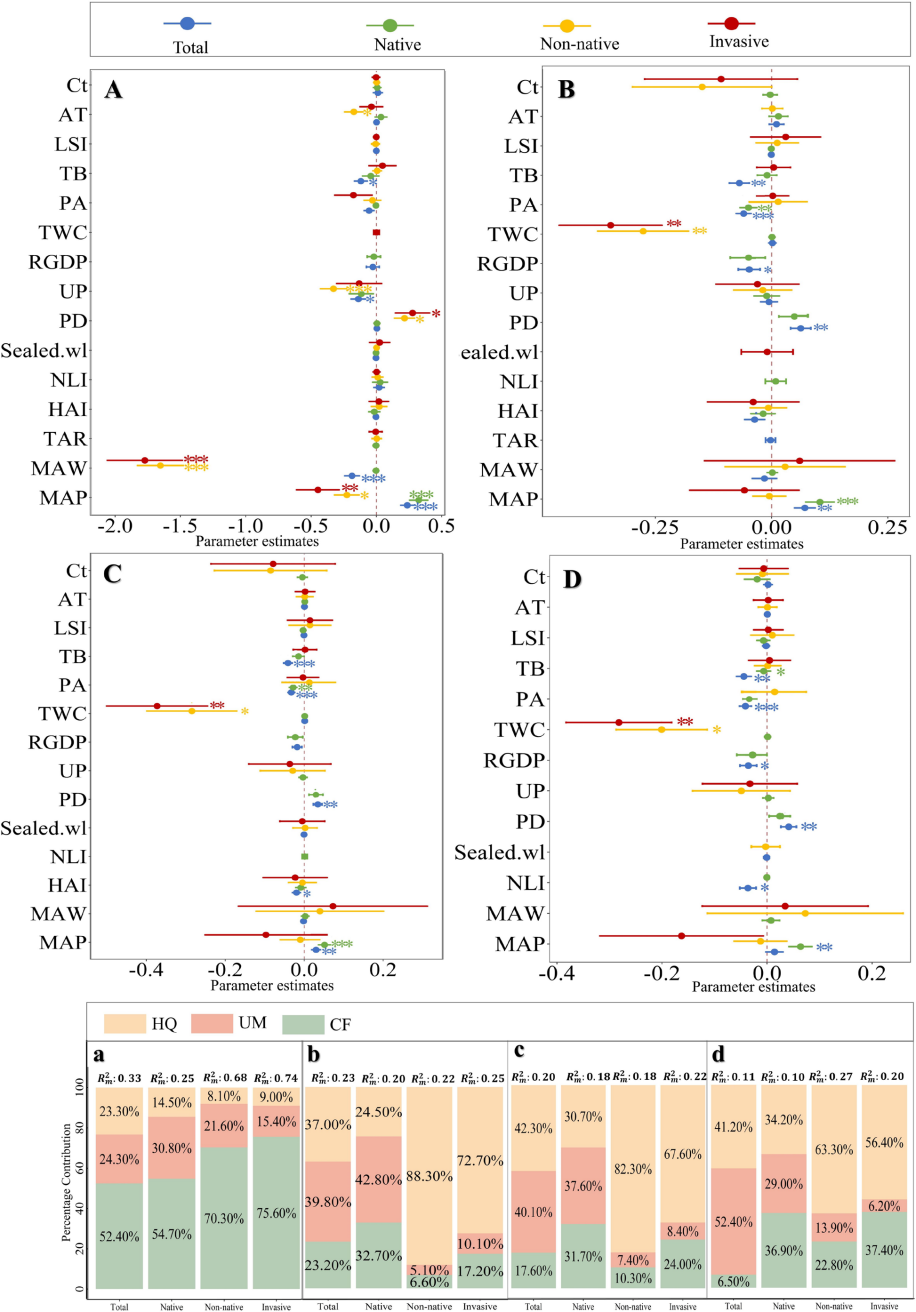
Effect and variance decomposition of climate factor (CF), urbanization metrics (UM), and habitat quality (HQ) on plant diversity indices of different origins (total, native, non‐native, invasive). A and a—Richness; B and b—Shannon; C and c—Simpson; D and d—Pielou. Abbreviations can be found in Table 1.

Climate factors mainly explained the variance in species richness for all groups (≥ 52.4%), especially mean annual precipitation (MAP) and mean annual wind speed (MAW), which explained more than 50% of the variability. For the other three groups (Shannon, Simpson, and Pielou), the explanation was lower (≤ 37.4%) (Figure 5a–d). In particular, MAP was significantly and positively correlated (*p* < 0.05) with both total and native species richness, Shannon, and Simpson, and was not significant except for a significant positive correlation (*p* < 0.05) with the Pielou of native plants. However, MAP was significantly (*p* < 0.05) negatively correlated with the richness of non‐native and invasive species. In addition, MAW, on the other hand, was significantly negatively correlated (*p* < 0.05) with species richness for all three groups of total, non‐native, and invasive species (Figure 5A–D).

Urbanization metrics and habitat quality have emerged as the predominant factors of plant diversity, collectively explaining over 60% of the variation in the Shannon, Simpson, and Pielou indices across all groups (Figure 5a–d). The specific environmental drivers influencing each diversity index varied among species groups. Regarding urbanization metrics, population density (PD) was found to positively significantly correlate with the three diversity indices of total and native, while night light intensity (NLI) and GDP per capita (RGDP) negatively significantly correlated with Pielou of total plants. Among habitat quality variables, total bare ground (TB) within each wasteland exhibited a significant negative correlation with all indices for total species (*p* < 0.05), suggesting that increased habitat disturbance and degradation are associated with reduced diversity and community evenness. Additionally, the patch area ratio (PA) was significantly negatively correlated with all three indices for both total and native species (*p* < 0.05), indicating that greater habitat fragmentation and irregular patch shapes adversely affect diversity, particularly for native flora. In contrast, for non‐native and invasive species, total woody plant cover (TWC) was the most influential habitat quality factor (56% explain power), with significant negative correlations observed for all three diversity indices (*p* < 0.05, Figure 5A–D).

**FIGURE 6:**
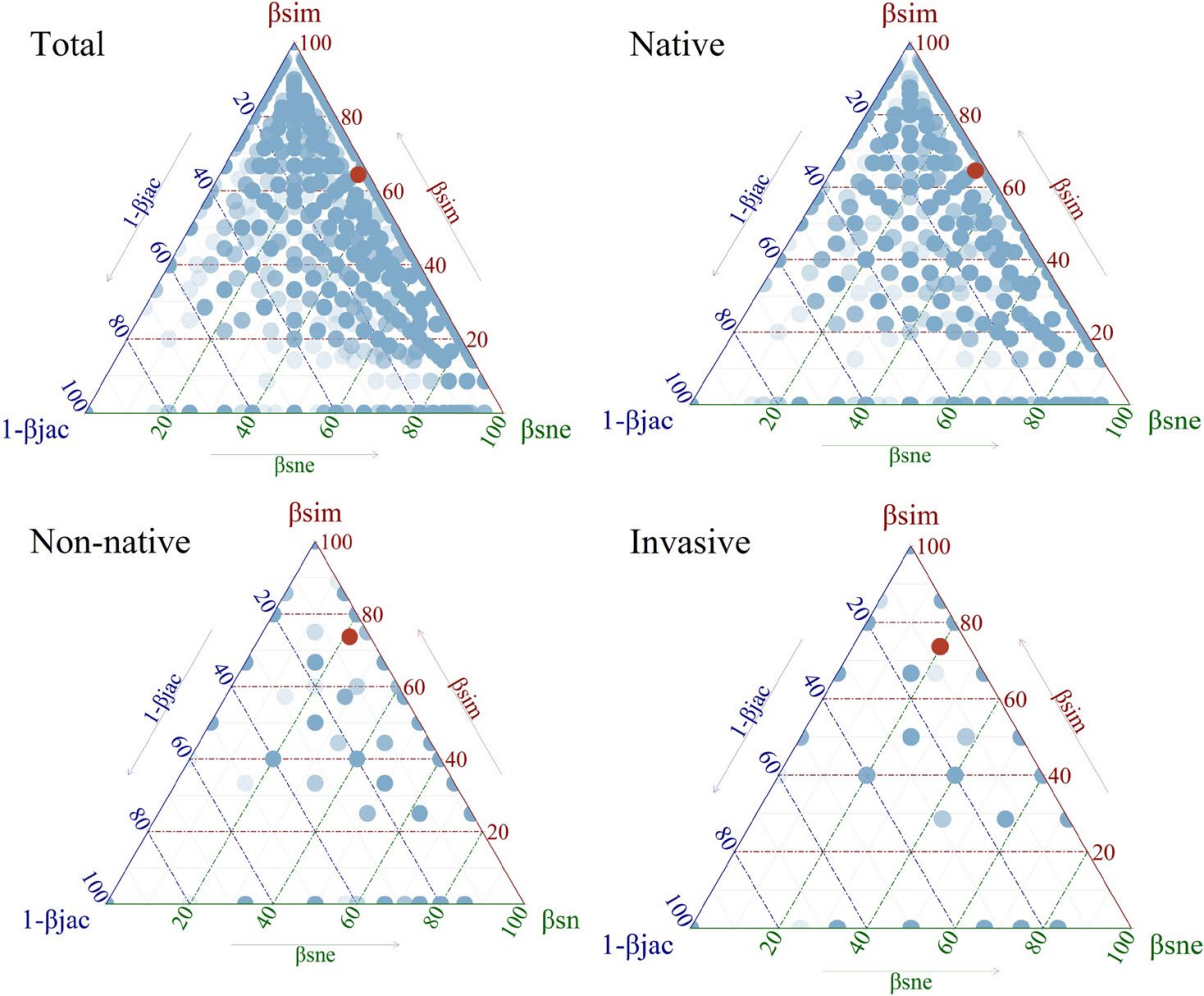
Decomposition of beta diversity in species communities of different origins (native, non‐native, and invasive). The red dot represents the average value of all sample points.

The result shows that most sample pairs cluster in the upper region of the triangle, near the *βsim* axis, indicating that overall community differences are primarily driven by species replacement components, while nestedness components (*βsne*) and 1‐*βjac* contribute relatively little (Figure 6). This implies that the overall spatial heterogeneity of native plant communities is primarily influenced by species replacement (*βsim*), reflecting that the differences between communities arise from factors such as environmental pressures and spatial environmental heterogeneity. Specifically, the mean points for non‐native and invasive species cluster closer to the *βsim* axis, indicating that the differentiation of non‐native and invasive species communities in urban wastelands on the QTP is significantly driven by local species replacement and environmental and historical heterogeneity.

**FIGURE 7:**
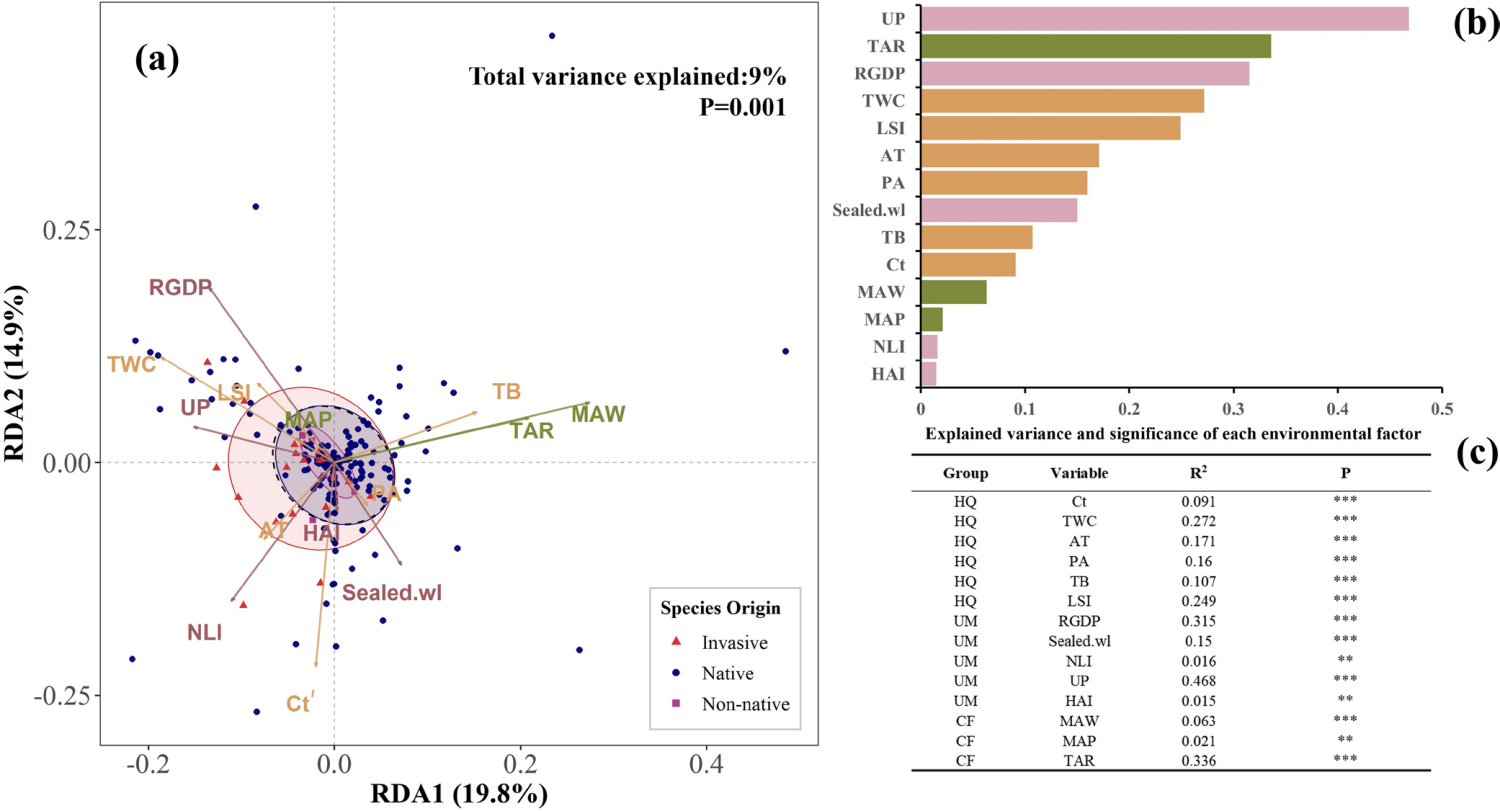
Relationships between species composition and environmental variables in urban wastelands on the QTP. (a) RDA ordination showing the distribution of native, non‐native, and invasive species along environmental gradients. (b, c) Relative contributions of individual environmental variables to the explained variance in species composition. In Figure (a), the dashed line represents the 95% confidence ellipse for all species, while the blue circle indicates native species, the red circle denotes invasive species, and the purple circle represents non‐native species. Abbreviations can be found in Table 1.

Different source groups exhibited distinct spatial patterns: total species and native species points clustered near the origin, indicating stronger adaptability; non‐native species and invasive species showed more dispersed distributions, jointly determined by urbanization and habitat quality factors, and were closely correlated with urbanization indicators (e.g., UP, RGDP) and habitat quality indicators (TWC, LSI). Redundancy analysis (RDA) indicates that climate factors (TAR, MAW, MAP), urbanization factors (RGDP, UP, NLI, Sealed.wl, HAI), and habitat quality factors (TWC, Ct, LSI, TB, AT, PA) significantly explain community composition (*p* = 0.001) (Figure 7a). All constrained variables collectively explain 9% of the total variance. Within the constrained variance, RDA1 and RDA2 accounted for 19.8% and 14.9%. Among these, urban population (UP, *R* ^2^: 0.47), annual solar radiation (TAR, *R* ^2^: 0.34), and GDP per capita (RGDP, *R* ^2^: 0.32) were the top three variables explaining community variation; Total woody cover (TWC, *R* ^2^: 0.27) and landscape shape index (LSI, *R* ^2^: 0.25) followed (Figure 7b,c).

## 4 Discussion

Urban wastelands function not only as seed reservoirs but also as ecological “stepping stones.” Embedded within highly urbanized matrices and often isolated by roads or built‐up areas (Linzhi; Figure 1), they store plant propagules that can disperse to surrounding green spaces via wind or animals, facilitating species exchange across fragmented habitats (Gao et al. 2025). In Linzhi, where urban areas are flanked by natural mountain ecosystems, the built environment may act as a dispersal barrier; in this context, wastelands can serve as intermediate habitats, promoting species movement between otherwise isolated patches.

This study provides a regional‐scale assessment of spontaneous plant diversity in urban wastelands across 17 cities on the QTP, a climatically extreme and ecologically fragile region. A total of 268 plant species were recorded, with a strong dominance of herbaceous taxa (Among them, herbaceous native plants account for the largest proportion). This pattern is jointly driven by the harsh high‐altitude environment—characterized by low temperature, aridity, and strong radiation—and the early successional stage of most sites. It is particularly evident in the Ali region (Cuoqin), where woody plants were rarely observed. In contrast, a certain proportion of woody species occurred in environmentally favorable areas (Linzhi and Changdu). Further analyses indicate that the occurrence of woody plants is influenced not only by regional climate but also by historical land use and soil conditions. For example, wastelands originating from green and open spaces (GOS) and land‐based water bodies (WB) tend to retain better soil structure and seed banks, facilitating woody plant establishment (Sun et al. 2025). Given that most wastelands are at early successional stages (< 10 years), similar to the study of the wasteland in Vieux‐Charmont (where herbaceous plants account for 75%), stable woody layers have not yet developed, resulting in herb‐dominated communities (Prach et al. 2014; Wang and Wang 2021).

In terms of community assembly, species richness was primarily driven by climatic variables such as mean annual precipitation, while diversity indices were mainly shaped by both urbanization and habitat factors. The differences between communities are primarily determined by species turnover influenced by environmental heterogeneity. By covering a broad environmental gradient encompassing varied climatic and urbanization intensities, the study enhances the generalizability of its findings and offers insights into plant community assembly across heterogeneous urban landscapes.

### 4.1 Native Dominance in Urban Wastelands of the QTP

The predominance of native species in urban wastelands on the QTP is consistent with previous findings (Brandl et al. 2004; Dai et al. 2025; Torija et al. 2025; Zhang et al. 2020). The proportion of native plants in Tibetan cities (85.8%) is notably higher than that reported for other regions of China, including Heilongjiang in the north (67.8%) (Zhu et al. 2024), and Yunnan (73.4%) (Gao et al. 2023), Chongqing (84.59%) (Huang et al. 2019), and Shenzhen (74.1%) (Liu et al. 2023) in the south, and in the more developed eastern region—Shanghai (82.5%) (Tian et al. 2014). This pattern underscores the resilience and ecological adaptability of indigenous flora in high‐altitude urban ecosystems (Gentili et al. 2024). The extreme environmental conditions of the QTP—low temperature, aridity, and high radiation—strongly constrain species establishment, favoring stress‐tolerant native taxa (*Suaeda corniculate* and *Ajania fruticulosa*) while limiting the persistence of introduced species. This filtering effect is particularly evident in remote cities such as Bange, Nima, and Cuoqin, where non‐native species are absent. Native species dominate plant communities across the QTP; widely distributed taxa such as *Artemisia sieversiana* and *Artemisia moorcroftiana* occur across most surveyed areas. In the eastern coniferous forest regions (Linzhi), communities are characterized by species such as *Clematis tenuifolia* and *Taraxacum tibetanum*. In contrast, in high‐altitude cold regions (Nima and Cuoqin), plant assemblages consist almost exclusively of native species (*Urtica hyperborea* and *Physochlaina praealta*). Desert regions in Ali Prefecture support distinct communities dominated by *Suaeda corniculata* and *Ajania fruticulosa*.

Although most cities on the QTP were established around the 1960s, their historical and cultural contexts continue to shape species composition. Socio‐cultural factors, especially in Tibetan Buddhist regions, may promote the introduction of non‐native species (e.g., ornamental or religious plantings), but such species rarely establish or spread under harsh alpine and arid conditions. For example, in areas such as Tashilhunpo Monastery, planted woody vegetation is still dominated by locally adapted species (*Salix paraplesia var. subintegra*), indicating that environmental filtering overrides human‐mediated introductions (Miehe et al. 2008). Conversely, some native species with cultural or religious significance may be closely associated with human activities and can contribute to spontaneous plant communities through escape or local dispersal (*Artemisia wellbyi*, commonly used for incense in Shigatse) (Salick and Moseley 2012). We frequently recorded cultivated species such as *Hordeum vulgare var. coeleste* in wastelands. As a staple crop widely grown across the QTP, its occurrence reflects local agricultural practices and land‐use patterns, which may further influence species composition and diversity in urban wastelands. In addition, these cities are characterized by small urban scale, low population density, and limited external connectivity, resulting in low propagule pressure, which further constrains the introduction and spread of non‐native species (Paul et al. 2020).

As well as the dominance of native species, several rare, endangered, and nationally protected plants were recorded. These species typically have narrow distributions, specialized niches, and low tolerance to disturbance, resulting in high conservation value (Mace et al. 2012). On the QTP, extreme environmental conditions have driven strong ecological specialization, but also limit regeneration capacity and resilience (Zhang et al. 2022). In this context, urban wastelands—characterized by relatively low disturbance—may serve as micro‐refugia for rare or low‐frequency native species, supporting their persistence and natural regeneration. Thus, they may play an important role in maintaining regional biodiversity and facilitating ecological restoration in high‐altitude urban systems (Petit‐Berghem et al. 2021).

Despite their low overall numbers, non‐native species exhibited high invasiveness (*Erigeron canadensis*, etc.), which is consistent with the results from Yunnan (51.4%). In all but three surveyed cities, invasive taxa accounted for over 79.1% of the non‐native flora, indicating a strong tendency for rapid spread and dominance once established. This may suggest a latent “invasion debt,” where a small pool of non‐native species has the potential for significant future expansion, particularly as urbanization and environmental changes intensify (Essl et al. 2011; Gao et al. 2023). Such invasive dynamics threaten the stability and integrity of native plant communities in urban wastelands. These findings underscore the need for both conservation and invasive species prevention, especially in ecologically sensitive and climatically extreme regions like the QTP (Salisbury et al. 2021).

### 4.2 Land‐Use Legacy Effects and Climatic Factors Drive Alpha Diversity in Urban Wastelands at the Regional Scale

Our findings demonstrate that differences in land‐use substrates of various origins, combined with climatic factors, particularly mean annual precipitation (MAP) and mean annual wind speed (MAW), collectively constitute the primary drivers shaping the diversity patterns of naturally formed plant communities in urban wastelands on the QTP. This region is characterized by its extreme natural environment, such as low precipitation, intense solar radiation, and persistent strong winds, which together create a formidable ecological filter.

Plant communities in wasteland exhibit pronounced variation in Richness, Shannon, and Simpson across land‐use types, whereas evenness remains invariant. This decoupling indicates that land‐use legacies primarily regulate community assembly by constraining species colonization and dominance hierarchies, rather than altering relative abundance distributions. Specifically, historical disturbances modify initial conditions and the strength of environmental filtering, thereby shaping the realized species pool (Cramer et al. 2008), while evenness is more tightly governed by biotic interactions and resource partitioning processes (Van der Plas et al. 2012). The convergence in evenness across land‐use types, despite divergence in diversity metrics, suggests that community assembly in these abandoned habitats is predominantly driven by deterministic processes. Under strong environmental constraints, stochasticity is reduced, and communities converge toward similar structural configurations (Chase 2007). This pattern is consistent with previous findings that urban systems impose relatively uniform environmental filters, leading to structural convergence among communities with contrasting land‐use histories (Sun et al. 2025).

Consistent with previous studies (Bradford et al. 2003; García‐Palacios et al. 2018; Wang et al. 2022), this pattern highlights the role of water availability, maybe as an important limiting factor of community assembly in arid and high‐elevation ecosystems (Wang et al. 2022). Abandoned waterbody (WB) habitats—which have evolved from former water bodies—maintain significantly higher species diversity. On the QTP, where water resources are generally scarce, water availability acts as the dominant ecological selection mechanism, shaping species coexistence patterns (Nicolas et al. 2021). Consequently, wastelands transitioning from aquatic systems likely retain higher soil moisture, expanding niche space and enabling the persistence of a broader range of species. Furthermore, we observed that increases in MAP significantly enhance the richness of native and total species, while simultaneously suppressing non‐native and invasive species. This pattern suggests that native species, having evolved under the plateau's harsh, variable conditions, may be highly responsive to even modest increases in moisture, quickly capitalizing on improved water availability to expand and persist. In contrast, non‐native and invasive species, which lack such local adaptations, are less able to exploit these episodic moisture pulses and are more likely to be excluded by environmental stressors (Liu et al. 2024; Petit et al. 2012). Thus, the plateau's unique precipitation regime acts as a strong environmental filter, favoring the persistence of locally adapted flora while limiting the establishment of exotics. In high‐altitude cold regions such as Ali, due to the extremely strong selective effects of the natural environment, no non‐native species were found in the flora of the surveyed wastelands. However, a significant number of native plant species representative of the region were present, such as *Chamaerhodos sabulosa*. Collectively, these findings demonstrate that the interaction between land‐use legacies and environmental filtering governs biodiversity patterns in urban wastelands, with water availability emerging as a primary constraint that can override the effects of land‐use history in water‐limited regions.

Our findings indicate that wind speed emerged as another crucial climatic factor influencing diversity, particularly at these high altitudes. This is consistent with relevant research (Momberg et al. 2021; Yu et al. 2025). Persistent strong winds can directly reduce species richness by causing mechanical damage, such as leaf tearing, flower and seed loss, or even uprooting, especially in species lacking specific adaptive traits (Julian and William 1986; Momberg et al. 2021; Yang et al. 2014). On the other hand, prolonged exposure to strong wind conditions usually closes the stomata of plants to reduce transpiration, which can cause the photosynthetic rate of the plant to decrease (Gardiner et al. 2016), which in turn causes the growth rate of the plant to decrease as well, affecting plant growth and reproduction (Bang et al. 2010). Moreover, high winds exacerbate soil moisture loss, further intensifying drought stress in shallow, poorly developed soils typical of the region (Fitzgerald and Kirkpatrick 2017). These combined effects not only limit the growth and reproductive success of many plants, but also reinforce the selective advantage of native perennials with drought and wind tolerance, while screening out less‐adapted exotics at early stages of colonization (Fahnestock et al. 2000; Zhang et al. 2007).

Our results show that wind speed has a significant negative impact on non‐native and invasive species, likely because, unlike native species, they have not evolved adaptive traits to cope with high‐altitude, high‐wind conditions, making them more susceptible to wind‐related stress (Alizadeh and Hitchmough 2019). More than 75% of the non‐native species we surveyed were found in low‐altitude areas with low wind speeds. For example, *Erigeron canadensis* (Invasion level 1) and *Erigeron sumatrensis* (Invasion level 1), although they are primarily wind‐dispersed and characterized by rapid spread over wide areas, their distribution in the wastelands of the QTP remains largely confined to lower‐altitude, low‐wind‐speed regions such as Maerkang and Ganzi. These species have not yet been recorded in high‐altitude, high‐wind‐speed areas such as Naqu and Ali. These findings highlight the role of local environmental filters, such as wind, in shaping community composition and resisting biological invasions.

### 4.3 Impact of Urbanization and Habitat Quality on Alpha Diversity

Urbanization intensity and habitat quality were identified as the primary determinants of plant community diversity in urban wastelands, collectively explaining over 60% of the variation in Shannon, Simpson, and Pielou indices across all groups. This finding aligns with a growing body of research highlighting the profound impacts of human activities and habitat quality on urban biodiversity patterns (Lepczyk et al. 2023; McKinney 2006). Urban environments, through land‐use change, habitat modification, and resource redistribution, fundamentally reshape the ecological processes that govern species assemblages and community structure (Seto et al. 2011).

Urbanization exerts complex and sometimes contradictory influences on plant biodiversity in urban wastelands. Our results show that higher population density is positively correlated with plant species richness, suggesting that increased human presence does not necessarily lead to biodiversity loss (Sushinsky et al. 2013). In many cases, human activities introduce additional resources, such as water and nutrients through sewage and organic waste, which can improve soil conditions and create heterogeneous habitats that support a greater variety of plant species (Meffert et al. 2012). On the QTP, where natural conditions are harsh, these anthropogenic inputs (the discharge of wastewater increases soil moisture, while the introduction of organic waste adds nutrients to the soil, etc.) may play a crucial role in facilitating the establishment and survival of spontaneous plants.

However, economic development, as reflected by GDP per capita (RGDP) and nighttime light intensity (NLI), tends to have the opposite effect on urban plant communities. High RGDP and strong nighttime illumination are typically associated with intensified urban infrastructure, industrial activity, and higher levels of anthropogenic disturbance. According to environmental filter theory, such disturbances selectively favor a narrow range of disturbance‐tolerant species, reducing community evenness and potentially increasing ecological vulnerability (Kohli et al. 2018; Petit et al. 2012). These findings highlight the potential ecological costs of unchecked economic growth and urban expansion.

Notably, the contrasting effects of population density and economic indicators reveal a decoupling between demographic and economic development in shaping urban biodiversity (Sushinsky et al. 2013). While higher population density may enhance species richness through resource enrichment and increased habitat heterogeneity, economic growth and urban intensification can increase disturbance and reduce species evenness (McKinney 2008). This divergence suggests that areas with dense populations do not always coincide with areas of high economic output or urban infrastructure, and their ecological impacts can differ significantly (d'Amour et al. 2017). Effective urban biodiversity conservation thus requires integrated strategies that address both direct human influences and the broader economic drivers of urban change, aiming to balance the benefits of increased species richness with the need to maintain community evenness and ecological resilience.

In the QTP region, habitat quality is the predominant factor shaping plant diversity within urban wastelands. Here, habitat quality is defined as an integrated measure of vegetation structure (e.g., TWC, TB), soil resource conditions (e.g., Ct), habitat size, and landscape configuration (e.g., PA, LSI), which together determine resource availability, niche heterogeneity, and disturbance intensity. Our findings reveal a significant negative correlation between the perimeter‐to‐area (PA) ratio and spontaneous plant diversity indices, echoing previous studies (Gao et al. 2023, 2021; Peng et al. 2019; Wang et al. 2020). As the complexity of the wastelands' edges (PA) increases, patch shapes become more irregular, and the relative length of edges expands, resulting in heightened edge effects. These edge zones often exhibit greater microclimatic variability and a wider range of habitat types, but they are also subject to increased disturbance intensity (Fahrig 2017; Gao et al. 2023; Moser et al. 2002). While a certain degree of edge complexity can promote habitat heterogeneity, excessive edge disturbance ultimately restricts the colonization and persistence of many native species, leading to an overall decline in plant diversity (Fahrig 2017; Gao et al. 2023; Moser et al. 2002). This pattern is supported by our field data. For example, in Linzhi, species richness reached a relatively high level at moderate PA (PA = 0.06, Richness = 47), whereas sites with higher PA showed markedly lower richness (PA = 0.22, Richness = 10).

For non‐native and invasive species, woody vegetation cover acts as a strong environmental filter, especially under the harsh conditions of the QTP. Among the cities we surveyed, Linzhi has the highest woody coverage (75%) among the wastelands. In contrast, wastelands such as Nima were dominated by herbaceous vegetation, with a woody vegetation cover of 0. Studies indicate that in habitats with high woody cover, species coexistence is shaped more by environmental filtering and stochastic effects than by competition (Kohli et al. 2018; Petit et al. 2012; Ren et al. 2022). Then, in the context of the QTP, woody cover serves as an indicator of habitat quality in landscapes characterized by high substrate exposure, intense radiation, strong winds, frequent freeze–thaw dynamics, and large diurnal temperature ranges. Dense woody vegetation modifies microclimate conditions by reducing ground exposure, retaining litter, and increasing fine‐root inputs. These processes improve soil structure, enhance organic matter and water‐holding capacity, and strengthen nutrient cycling (Barbier et al. 2008; Gong et al. 2021; Pilon et al. 2021; Zangy et al. 2021). Collectively, these effects narrow the establishment windows for heliophilous, fast‐growing invasive species while favoring conservative, shade‐tolerant native plants—thereby intensifying environmental filtering against invasion. Most invasive species are r‐strategists, typically adapted to high‐light and disturbed habitats (Grime 2001). For example, *Erigeron canadensis* and *Erigeron sumatrensis* are all light‐demanding species that primarily occur in open habitats such as wastelands, field margins, and roadsides. Their high photosynthetic capacity and rapid relative growth rates are optimized in open, sunlit environments (Van Kleunen et al. 2010), making them less competitive under dense woody canopies. In our survey, non‐native species were dominated by members of the Asteraceae family, which are well‐known pioneer plants with strong reproductive and dispersal abilities (Schuster et al. 2018). The light‐demanding and disturbance‐adapted traits of these species further explain their reduced abundance under conditions of higher woody cover. Additionally, greater woody cover is associated with later successional stages and more stable habitats (Ren et al. 2022). Such environments experience fewer and weaker disturbances, thereby limiting the recruitment opportunities for early‐successional invaders and enhancing priority effects that favor established native species. Furthermore, the structural complexity provided by trees and shrubs attracts birds, insects, and other fauna (Karen et al. 2012; Long and Frank 2020), promoting seed dispersal and ecological interactions that strengthen native community resilience.

### 4.4 Species Turnover Dominates the Overall Community Diversity

Consistent with previous findings, our results show that the dominant role of species turnover (*βsim*) in overall community differentiation indicates that environmental heterogeneity and spatial succession are key drivers of plant community composition in these urban wastelands (Qian et al. 2020; Yao et al. 2023). This aligns with broader ecological theories, where species turnover often reflects niche differentiation and adaptation to diverse environmental conditions (Gleason 1939; Wen et al. 2024). The mean points for alien and invasive species cluster closer to the *βsim* axis, indicating that their community differentiation within urban wastelands is particularly influenced by local species replacement and environmental heterogeneity (Soininen et al. 2018).

The results of the Redundancy Analysis (RDA) in this study indicate that the selected climate, urbanization, and habitat quality variables significantly explain differences in urban wasteland plant community composition (*p* = 0.001). Among these, population size (UP, *R* ^2^: 0.47), total annual solar radiation (TAR, *R* ^2^: 0.34), and GDP per capita (RGDP, *R* ^2^: 0.32) were the strongest drivers, indicating that urbanization intensity and natural energy input levels significantly influence community variation (Güneralp and Seto 2013). Hou, Shi, et al. (2023) and Hou, Li, et al. (2023) noted that urbanization indicators (e.g., population density, economic output) are primary drivers of alien plant abundance (Hou, Li, et al. 2023). This study further reveals that tree‐wood canopy cover (TWC, *R* ^2^: 0.27) and landscape shape index (LSI, *R* ^2^: 0.25) also significantly influence community structure, suggesting that habitat structural complexity exerts a pronounced regulatory effect on plant composition (Fan et al. 2017). Higher woody plant cover typically indicates environmental stability and low disturbance levels, which are conducive to the maintenance of native species (Aronson et al. 2014). For example, in Linzhi, native species richness was relatively high (25 species) on wasteland with the highest forest cover (75%); whereas in wasteland with lower forest cover (15%), native species richness was relatively low (9 species).

From the pattern of community composition, the RDA plot shows that native species points are clustered and close to the origin, indicating their strong tolerance to environmental changes or broad ecological niches (Zhang et al. 2023); non‐native and invasive species points are more dispersed and show a clear shift along the urbanization gradient (aligned with arrows for UP, RGDP, Sealed.wl, etc.), indicating their distribution is more influenced by urbanization and human disturbance (Liu et al. 2025). This spatial segregation pattern supports the “dispersal pressure + environmental screening” hypothesis: densely populated areas and regions with high economic activity exhibit higher probabilities of introducing alien species (Fang et al. 2024; Lockwood et al. 2005). Furthermore, the artificiality of habitats in these areas—such as high surface sealing and reduced woody vegetation—further screens for disturbance‐adapted “r‐strategist” plants (Grime 2001).

### 4.5 Conservation Implications

Our findings demonstrate that conservation strategies for high‐elevation cities must account for the dominant role of biogeographic processes in shaping urban plant diversity (Jiang et al. 2022). Climatic filtering associated with precipitation and wind speed acts as a strong constraint on regional species pools. Additionally, understanding the filtering effects of woody vegetation and microclimate on community assembly may help managers design more resilient, biodiverse, and invasion‐resistant urban landscapes, even outside the context of wastelands themselves. For example, in areas where natural conditions permit the growth of woody plants, when planning urban green spaces, the selection of woody plants should prioritize native species of a certain height (D'Antonio et al. 2024). This can reduce light levels in the groundcover layer through shading, thereby suppressing invasive herbaceous species or pioneer species that thrive in high‐light conditions (Kaur et al. 2024). Then, we should also consider that in most high‐altitude cities, woody vegetation is naturally limited by climatic and edaphic constraints, and many urban wastelands are unsuitable for deliberate tree planting due to poor soil development, strong radiation, and shallow substrates (Smith 2013). In this context, woody cover should be interpreted as an indicator of natural successional progression rather than a management target. Therefore, instead of artificially increasing woody vegetation, urban ecological planning should aim to preserve and protect later‐successional wastelands where native woody or semi‐woody species have already become established (Niu and Cai 2024). In the high‐altitude regions of the Ali, it is particularly important to conserve wastelands that have reached relatively late successional stages. For example, in Ali, a wasteland with an abandonment duration of over 10 years supports late‐successional communities, including *Krascheninnikovia ceratoides* and *Ajania fruticulose* communities. These communities are representative of the region's natural vegetation and play an irreplaceable role in conserving regional biodiversity. Such sites represent stable habitat patches with higher ecological resilience and stronger natural resistance to invasion. For earlier‐successional or sparsely vegetated wastelands (abandonment time < 2 years), management efforts should focus on minimizing new disturbances, such as soil excavation or construction activities, and on reducing propagule pressure from nearby invasive sources (e.g., horticultural escapes or transport corridors). Maintaining a mosaic of successional stages across the urban landscape can enhance overall biodiversity while naturally constraining invasion processes.

Meanwhile, conservation strategies should be more systematically integrated with local cultural practices and traditional ecological knowledge. In QTP cities, culturally influenced spaces—such as areas surrounding monasteries, prayer paths, and traditional settlements—are typically subject to low disturbance and tend to favor the retention of native species that are culturally valued or well adapted to local conditions (Fu et al. 2026; Salick and Moseley 2012). This culturally mediated management promotes the persistence of native plants and acts as a “cultural filter” limiting the introduction and spread of non‐native species. Within urban landscapes, such spaces can function as refugia for native biodiversity. Incorporating similar low‐disturbance management regimes and prioritizing native species in culturally sensitive areas may help enhance the stability and ecological integrity of urban plant communities. (Fu et al. 2026). In summary, from the perspective of urban ecological management, it is crucial to control transmission pathways (such as restricting horticultural introductions and logistics‐related dispersal), increase the coverage of native woody vegetation, systematically integrate into the local culture, and maintain habitat connectivity. These measures help mitigate the spread of invasive plants while promoting the stability of native plant communities and ecological restoration.

In summary, the findings from urban wastelands on the QTP provide valuable ecological insights that can inform the management of other urban green spaces in similarly harsh and sensitive regions. The strong dominance and resilience of native species, the role of climatic and habitat filters, and the mechanisms limiting biological invasions all highlight the importance of maintaining habitat heterogeneity and supporting natural succession processes in urban green space planning. For the green spaces that will be deliberately planned and constructed in future urban, strategies such as preserving native plant communities, enhancing habitat quality, and minimizing unnecessary disturbances can draw on the ecological lessons from wastelands.

### 4.6 Limitations and Prospects

Although our sampling strategy was designed with strict criteria to encompass cities with diverse climatic conditions and developmental stages, and included wastelands of varying sizes and abandonment durations, differences in the number of accessible wastelands among cities were unavoidable (Petit‐Berghem et al. 2021). In some cities, the total number of wastelands was relatively small, while in others, field access was restricted for certain sites. Consequently, the number of sampled wastelands varied across cities. To minimize the potential influence of this unevenness on our results, we incorporated key environmental and urban variables—such as built‐up area, population size, impervious surface ratio surrounding each wasteland, and total wasteland area—into our analyses to represent ecological and land‐use differences among cities. We acknowledge this as an inherent limitation of large‐scale urban ecological surveys. Future research could address this issue more systematically by improving spatial comparability and data completeness through measures such as expanding sample sizes, enhancing accessibility to study areas, and incorporating time‐series data. Furthermore, the observed plant diversity may integrate both legacy effects from historical propagule pools and current responses to climate, urbanization, and habitat structure, and disentangling these pathways would require seed bank surveys or temporal monitoring (Godefroid et al. 2007; Xia et al. 2024). Future studies should explicitly evaluate seed bank contributions—through germination experiments or seedling emergence assays—to more robustly partition the relative influence of historical versus contemporary drivers on wasteland plant diversity. This acknowledgment enhances the transparency of our causal inferences and highlights an important avenue for mechanistic follow‐up research on the QTP. Although *β*‐analysis results indicate that climate, urbanization, and habitat factors do play a role in shaping community structure, within such an extensive research area characterized by extreme spatial heterogeneity, most community differences likely still stem from random processes, spatial structure, or unmeasured environmental factors (Hubbell 2011). In addition, future studies could adopt a socio‐ecological perspective to examine how residents perceive plants of different origins—invasive, non‐native, and native species—and to explore the mechanisms driving their preferences. Such research could also develop more effective approaches to account for the edge effects of surrounding detailed land‐use types around wastelands, which may strongly influence plant community composition and dynamics.

## Author Contributions

**Lin He:** conceptualization (lead), data curation (lead), formal analysis (lead), investigation (equal), methodology (lead), validation (lead), visualization (lead), writing – original draft (lead), writing – review and editing (lead). **Zhiwen Gao:** conceptualization (supporting), data curation (supporting), formal analysis (supporting), investigation (equal), methodology (supporting), supervision (equal), validation (supporting), visualization (supporting), writing – review and editing (supporting). **Yao Yao:** data curation (supporting), investigation (equal), writing – review and editing (supporting). **Luyi Lan:** data curation (supporting), investigation (equal), writing – review and editing (supporting). **Xiaoya Yu:** investigation (equal), writing – review and editing (supporting). **Yanyi Yang:** data curation (supporting), writing – review and editing (supporting). **Xinyi Luo:** data curation (supporting), writing – review and editing (supporting). **Ruishen Yang:** data curation (supporting), investigation (equal), writing – review and editing (supporting). **Junwei Wang:** investigation (supporting), writing – review and editing (supporting). **Yuandong Hu:** project administration (supporting), writing – review and editing (supporting). **Qiong La:** investigation (supporting), writing – review and editing (supporting). **Liangjun Da:** funding acquisition (lead), investigation (supporting), project administration (lead), resources (lead), supervision (equal), writing – review and editing (supporting).

## Funding

This research was funded by the Special Project of the *Flora of China* (2015FY210200‐4), under the Ministry of Science and Technology of the People's Republic of China.

## Conflicts of Interest

The authors declare no conflicts of interest.

## Supporting information


**Table S1:** Basic information on the cities surveyed.

**Table S2:** Basic information on the wastelands surveyed.

**Data S1:** ece373521‐sup‐0003‐DataS1.rar.

## Data Availability

All data and code used have been submitted in Supporting Information and are publicly available, ensuring full reproducibility.
